# Annotations

**Published:** 1888-09-22

**Authors:** 


### Annotations.

**Fresh Fruit as Food.**

The human organism is a laboratory for the transformation of various kinds of food into various kinds of energy. A man is a sort of chemical crucible, who receives all kinds of things into his interior as food, and turns them into a great variety of forces—for example, muscular force, nerve force, thought force, and so on. Without food the man can make no force at all; with insufficient food he will make insufficient force; with unsuitable food he will make defective and unsuitable force. That is as exactly and precisely true as that two and three makes five. The power to run is a product of food and the man, the power to use the teeth and to digest is a product of food and the man, the power to breathe is a product of food and the man, the power to think is a product of food and the man. Deprive the man of food, and all these powers will promptly "cease and determine," as the lawyers say. Food, then, is a circumstance of the very primest importance. As a man eats so will he breathe, run, digest, think. It is impossible to exaggerate the importance of the food question. A Royal Commission of all the ablest physicians and scientists in the world could have no more fitting employment than the investigation of the question of human food. Obviously, we cannot deal with such a subject exhaustively in a mere "Annotation." We can, however, express a decided conviction as to the value of fresh ripe fruit as food. Apples, pears, plums, apricots, peaches, gooseberries, grapes, and all these fruits, when fresh and ripe, stand at the very summit of excellence as human foods. They fulfil the essential conditions of pleasantness, digestibility, nutriency, and medicinality. For summer eating they are especially admirable, because they are both satisfying, thirst-quenching, and light. For those who are constipated they are excellent substitutes for pills and mineral waters. We ought to eat, as a people, at least ten times the fruit we do. But we cannot get it. True; but that is our own fault. The recently-held National Congress of Fruit Growers unanimously agreed that we have one of the finest climates and some of the best soils in the world for fruit growing. They also agreed that fruit could be produced at a quarter of the price which it now often reaches, and that thus it could be placed within the reach of all. What we want is a better system of land tenure, with greater railway facilities. These could be easily secured if the English were a people of brains and purpose. Being, however, dull and stupid, and exceedingly well pleased with ourselves, we must still continue to pay sixpence instead of a penny each for "William" pears, and six shillings a pound for hothouse grapes; and we must also do as we have done before, take nauseous pills and mineral waters for the benefit of stomach and bowels, instead of delicious and ten times more health-giving fruit. "Truly," as we often say in these pages, "we are a clever and far-seeing people."

**The Battle of the Diets.**

Vegetarians whether pure or only partial, often express themselves in language which cannot be scientifically justified; nevertheless by their words and works they compel attention to a subject of the first importance. Should man eat flesh or vegetables? that is the question. "Vegetables," say the vegetarians, and they give a hundred and fifty reasons which to themselves, at any rate, are convincing beyond the possibility of refutation. Flesh-eaters, on the other hand, as stoutly maintain that for them, at least, beef and mutton, with an occasional flight into the regions of the farmyard, the woods, and the moors, are essential to health. A recent correspondent of a daily newspaper affirms that he has tried vegetarianism in the thoroughest possible manner; he made six separate trials, extending, with intervals, over eleven years; and he is convinced, as the result of his experiments, that the dietary is not only a delusion but an injury and a danger of the most serious kind. He knows of at least two who died in "the very prime of beautiful and useful lives, martyrs to vegetarianism." Speaking as a medical man, the present writer is bound to express the conviction that vegetarianism pure and simple is not suited to man *in these islands*. The last three words have an important bearing on the controversy, and perhaps, indeed, they are the true key to the secret. He believes that he has seen one man of forty-four who but for vegetarianism would have been alive and comparatively well at the present time. It must be understood that the question is one which can only be settled by experience, not by appeals to scientific generalisations. Two considerations will make this clear. The vegetarians affirm that man is not by nature a flesh eater because he has not the teeth of the typical carnivore. That is true. But the flesh eater may as properly reply that he is not by nature a vegetable feeder because he has not the stomach of the typical graminivore. The tiger is a type of the flesh eater, and his teeth differ markedly from those of man; but, on the other hand, the ox is an equally typical graminivore, and his stomach differs much more from that of man than do the teeth of the tiger from those of his human fellow mammal. Experience, then, must settle the question both for the race and for individuals. How very generally experience has settled it, and settled it with sense and wisdom! In warm countries, where the blood is hot, men do not need so stimulating and sustaining a diet as they do in colder climates. In these countries fruits and vegetables generally abound, and are obtainable with a minimum of labour. The natural, and indeed inevitable result, is a very general prevalence of vegetarianism. In colder countries, where the blood requires a more heating diet, and where labour requires a more sustaining one, flesh eating is as generally necessary and as generally prevalent. There is the whole case in a nutshell. The common sense of all has settled the question of diet as it has settled that of clothing, of poetry, of religion, and of a thousand other things.

**Specialists Again.**

A contemporary reports the case of a lady who, feeling ill, had diagnosed her own diseases. With the sublime wisdom of ignorance she came to the conclusion that she suffered from affections of four different organs—heart, liver, nerves, and eyes. Accordingly she consulted a "specialist" for each disease, with the business-like intention of going to the fountain head at once, and getting cured without loss of time. But to her astonishment and disgust she did not get better at all, and finally was obliged to go back to the family practitioner. Our contemporary thereupon falls foul of both specialists and family doctors, more than hinting that the former are arrant humbugs and the latter lazy knaves. He admits a suspicion, however, that the public may be as much to blame as the specialists, in that they display such an unreasoning and ever-increasing faith in specialism. This last suspicion is amply justified by facts. Every doctor who has known what it is to be a "rising young man," either in family or consulting practice, has been asked, both by zealous friends anxious to do him a service and by strangers consulting him for the first time, what his specialty was? The disappointment of both friends and strangers has been so evident when they have been informed that no claim to specialism was made, that many a man has been severely tempted to assume a specialty for the mere sake of more rapid success in practice. What is to prevent any average medical man's calling himself a specialist for the liver, the spleen, the kidney, the bowel, or any other organ which is out of sight, and which the public know nothing at all about? Of course, for a man really to yield to such a temptation is to sink at once into the ranks of knavery and scoundrelism. He becomes immediately a fraudulent thief, as fit for prison as any other felon who obtains money by false pretences. Similarly there is a great demand by the public for practitioners who have written books. To write a book is the orthodox way of advertising yourself into practice. Your friends advise you to "read up" a subject and write upon it, sometimes offering to defray the cost of publication. "Merciful Heavens!" as Carlyle would have said, is not the world already sufficiently flooded with the stolen goods of medical compilations already? Nowadays, the medical man really honours himself who refrains from writing books. If a physician has had a large and varied experience, or has seen uncommon cases, it may be his duty in either event to publish what will be of service to his fellows; but to "read up" everything that has ever been written on a given medical topic for the mere sake of compiling a book is to injure medical science, and to sink the writer to the level of Beecham or Holloway. Specialism and book compiling are in many instances the most flimsy devices for escaping from the honest but severe and ill-paid work of family practice. Those who have trustworthy and capable family attendants will do well to trust them entirely, and to see neither specialist nor consultant without their concurrence.

